# Profile of international air passengers intercepted with illegal animal products in baggage at Guarulhos and Galeão airports in Brazil

**DOI:** 10.1186/2193-1801-3-69

**Published:** 2014-02-06

**Authors:** Cristiano Barros de Melo, Marcos Eielson Pinheiro de Sá, Flaviane Faria Alves, Concepta McManus, Lucas Fernandes Aragão, Bruno Benin Belo, Paulo Ricardo Campani, Antonio Cavalcanti da Matta Ribeiro, Christina Isoldi Seabra, Luiza Seixas

**Affiliations:** Campus Darcy Ribeiro Asa Norte, ICC Sul, Universidade de Brasília (UnB/FAV), ZIP Code 70.910-970 Brasília, DF Brazil; International Agriculture Surveillance (VIGIAGRO) - Ministry of Agriculture, Livestock and Food Supply (MAPA), Brasília, Brazil; International Agriculture Surveillance (VIGIAGRO) - Ministry of Agriculture, Livestock and Food Supply (MAPA), Galeão Airport, Rio de Janeiro, Brazil; International Agriculture Surveillance (VIGIAGRO) - Ministry of Agriculture, Livestock and Food Supply (MAPA), Guarulhos Airport, São Paulo, Brazil

**Keywords:** Airport, Baggage, Illegal food, Infectious disease, Passengers, Zoonosis

## Abstract

Protection against biological material entering a country or region through airports is important because, through them, infectious agents can quickly reach exotic destinations and be disseminated. Illegal products of animal origin may contain hazardous infectious agents that can compromise animal and public health. The aim of this study was to identify associations between possession of illegal animal products in baggage and demographic characteristics of the passengers, as well as characteristics of their travel plans in the two main Brazilian international airports. A total of 457 passengers were divided into two groups: passengers identified as carrying illegal animal products and control. Passengers identified as carrying illegal animal products not stated on the accompanied baggage declaration completed a questionnaire, to aid in profiling. Nationality, origin, age and residency of passengers were analyzed using chi square, logistic regression and odds ratios. Passengers from Eastern Europe were the most likely to enter with animal products as were those aged between 35 and 55 years. When evaluating the departure point, the highest frequency was seen in those coming from Portugal. Passenger group, reasons for travel, amount and type of baggage were available only for passengers identified as carrying illegal animal products, noting that they prefer traveling alone, for leisure, bringing few bags. Such information can contribute to the early identification of passengers that have illegal animal products in baggage at Brazilian airports.

## Background

Airports are the most important country frontiers to be protected because infectious agents can reach destinations and disseminate very quickly and they are strategic areas for national veterinary surveillance. This is because infectious agents can be transported in food of animal origin, especially those without health certificates and these can be detected in international baggage (Taniguchi et al. 2008Chaber et al. 2010) using X-ray scanners. Airborne, food-borne, vector-borne, and zoonotic infectious diseases transmitted during commercial air travel became an important public health issue due to the greater affordability of air travel, increasing the mobility of people (Mangili and Gendreau 2005). To minimize the risk of these diseases entering a country, products of animal origin need to be accompanied by an international health certificate. This is crucial to assure a reliable health situation when countries with known health status are involved, as regulated by the World Organisation for Animal Health (OIE) for its member countries (Pastoret and Chaisemartin 2011).

The International Agricultural Surveillance of the Ministry of Agriculture, Livestock and Supply (VIGIAGRO/MAPA) has the authority to check the entry of all agricultural products into Brazil. In Brazil all animal products or by-products, genetic material (eggs, embryos, semen etc.) without an international health certificate should be seized and destroyed. With the recent increase in international travel, these authorities have to be efficient in identifying passengers bringing products of agricultural origin into Brazil (Brazil Ministério da Agricultura, Pecuária e Abastecimento 1998). Preliminary studies have shown that about 39 tonnes of illegal animal products were seized from airline passengers in a four year analyses at Guarulhos International Airport (2006 to 2009) and about 19 tonnes in a two year analyses (2008 to 2009) from Galeão International Airport in a retrospective study (unpublished data).

A serious outbreak of African Swine Fever occurred in Brazil in 1978, and caused huge economic losses to the country before it was eradicated in 1984. Intensive epidemiological work was carried out by researchers from various Brazilian and international institutions, involving a number of public authorities. Brazil declared an Animal Health Emergency and adopted immediate measures to contain and eradicate the disease, following recommendations of OIE. The owner of the farm was an officer stationed at Galeão International Airport and he had collected leftovers from meals served on international flights to feed to his animals, including food from Portuguese and Spanish airlines. The direct and indirect costs of emergency actions reached US $ 13 million, including compensation for the loss of income from the slaughter of 66,902 pigs. Thus, emergency action also caused unemployment in 2,000 families who depended on pig farming in the region (Moura et al. 2010).

Studies conducted by the Department of Animal Health of Brazil identified the entry of 139 new diseases, infectious agents or vectors in the period from 1811 to 1980. These diseases included: Classical Swine Fever in 1866, Foot and Mouth Disease (FMD) in 1895 and Rinderpest in 1921. During this period, every 15 months a new disease or nosological agent entered Brazilian territory. This was compounded in the late 1970s, with the notification of 27 new diseases, including African Swine Fever. It is believed that this growing trend occurred due to the greater vigilance by the veterinary services that were structured in most states during this period and the consequent increase in health surveillance (Moura et al., 2010).

Brazilian Gross Domestic Product depends on its livestock industry as the country is a major exporter of beef to the European Union (ABIEC Associação Brasileira das Indústrias Exportadoras de Carne 2012; Carvalho et al., 2014), has the largest commercial cattle herd in the world, with approximately 195,5 million of animals in 2009 (IBGE Instituto Brasileiro de Geografia e Estatística 2013) and is the global leader in beef exports (Brazil Ministério da Agricultura, Pecuária e Abastecimento 2013). Also, Brazil has been the world’s largest exporter of poultry meat since 2004, as well as being the third largest producer, behind only the USA and China, and exports poultry to more than 150 countries (Brazilian Chicken 2014).

Upcoming major sporting events to be held in Brazil, such as the FIFA World Cup (in 2014) and the Olympics (2016), will significantly increase passenger movement, as well as the risk of introduction of infectious agents through airports. Measures need to be taken, therefore, to restrict the entrance of infectious agents into the country in baggage of international air passengers. To get an idea of the scale of the increase in movement during the FIFA World Cup, between June and July 2014 the National Civil Aviation Agency has authorized Brazilian airlines to operate 1,973 new flights in this short period, and authorized changes in approximately 80,000 existing flights. The number of created and modified flights will impact on about 42% of the Brazilian airline network (ANAC National Civil Aviation Agency of Brazil 2014).

The expansion of veterinary surveillance in international airports is a priority, to prevent the introduction of infectious agents that may compromise the health of livestock. Therefore, a profile (description of distinguishing features people or groups of people) of international air passengers that enter Brazilian territory with animal products is needed. The aim of this study was to identify associations between possession of illegal animal products in baggage and demographic characteristics of the passengers as well as characteristics of their travel plans in the two main Brazilian international airports.

## Methods

Official information was collected at the two main international airports of Brazil. São Paulo/Guarulhos - Governador André Franco Montoro International Airport (Guarulhos) and Rio de Janeiro/Galeão - Antonio Carlos Jobim International Airport (Galeão) are the two busiest airports in terms of number of international passengers in Brazil, respectively. Guarulhos is also the busiest cargo airport in Latin America. These two airports represent about 85% of international passenger arrivals to Brazil (Infraero Empresa Brasileira de Infraestrutura Aeroportuária 2012).

The present study had its technical and ethical procedures approved by the National Council for Scientific and Technological Development (CNPq) through process number 578255/2008-1 and special permits were obtained from the General Coordination of International Agricultural Surveillance of the Ministry of Agriculture, Livestock and Supply (VIGIAGRO/MAPA - number 294/2010) as well as the Federal Revenue of Brazil (number 00571/2009).

After landing, baggage from randomly selected airplanes was inspected noninvasively using an X-ray scanner and those with organic products were intercepted by a representative of MAPA/VIGIAGRO. Passengers were intercepted by the official service, according to Brazilian standard protocols (Brazil Ministério da Agricultura, Pecuária e Abastecimento 2006), from 119 international flights of 35 air companies in these two airports. Passengers were divided into two groups: passengers identified as carrying illegal animal products and control. Passengers found carrying animal products (milk, cheese, meat, salami, sausage, fish etc.) on international flights arriving at Guarulhos and Galeão International Airports were considered “passengers identified as carrying illegal animal products”. An officially approved questionnaire was used and all passengers were interviewed by a single researcher. Information was collected concerning the provenance, origin, nationality, type of baggage, amount of baggage, who the passenger was travelling with, reason for travel, sex and age of the passengers using a questionnaire that also contained information from the notice of seizure. Passengers that had their baggage inspected by X-ray machines and had no animal products were considered control. These passengers also had their DBA’s (Accompanied Baggage Declaration - IRS) analysed. DBA’s contained data on the provenance, origin, nationality, age and sex of these passengers. This procedure was carried out on twelve occasions from April 23, 2010 to August 19, 2011 (six missions each in Guarulhos and Galeão International Airport).

Information contained on official forms filled by the MAPA inspector at the time of interception of passengers carrying illegal animal products in their baggage were also analysed. No information that could violate privacy of passengers was obtained or used in this study. Also, the information of the control group was obtained from officially approved forms (DBA - Federal Revenue of Brazil).

All passengers signed a notice of seizure of illegal products in their baggage. Following standard procedures, passengers who had illegal products seized were released and did not pay any fines or fee.

The minimum number of passengers was calculated as 132 interceptions (minimum + 10% = 120 + 12 = 132; n = 120 when z = t) according to Sampaio (2010), and this “n” was defined to estimate the confidence intervals of the values found. One hundred forty-nine (149) passengers identified as carrying illegal animal products were intercepted at Guarulhos International Airport and 111 passengers at Galeão International Airport, totaling 260 passengers. One hundred ninety-seven (197) passengers served as the control group, 131 in Guarulhos International Airport and 66 in Galeão International Airport.

Data were analyzed using SAS® (Statistical Analysis System, v.9.3, Cary, North Carolina). Chi-square (*χ*2) tests and logistic regression analysis were carried out to determine differences between and within the two groups of passengers. ROC (Receiver Operating Characteristic) curves were produced and odds ratios calculated. Countries were grouped into regions or continents to facilitate the analysis. This grouping was based on historical and cultural rather than necessarily geographical ties.

Africa: South Africa, Angola, Egypt, Morocco.

North and Central America: Canada, Costa Rica, Cuba, United States, Mexico, Panama, Puerto Rico.

South America: Argentina, Bolivia, Chile, Colombia, Peru, Uruguay, Venezuela.

Asia: China, Korea, India, Japan, Taiwan.

Brazil: Brazil.

Latin Europe: Spain, France, Italy, Portugal.

Eastern Europe: Romania, Turkey.

Oceania: Australia.

Western Europe: Germany, Holland, Hungary, Norway, Poland, United Kingdom, Switzerland.

Middle East: Qatar, United Arab Emirates, Israel, Iran, Iraq, Israel, Lebanon, Turkey.

Russia: Russia and its borders Lithuania and Ukraine.

Ages were also divided into four groups: Group 1 less than 20 years, group 2 between 20 and 35 years, group 3 between 35 and 55 years and group 4 more than 55 years.

## Results and discussion

In this study, when the baggage was opened after interception, animal products were found in all of them, showing that the scanner operator identified animal products in the bags correctly. One hundred ninety-five (195) dairy (milk sweets, liquid milk, milk powder, condensed milk, yoghurt and cheese), 160 meats (ham, bologna, sausage, sausages in general) of cattle, buffalo, goat, chicken, llama, kudu, sheep, pig and unidentified species origins (usually packages in languages not identified by staff such as indigenous dialects), and other products such as honey, eggs and exotic pet products, which totaled 657.4 kg were seized from the passengers in the present study.

Significant effects of month, origin, nationality, airport, provenance and age were seen between the two groups of passengers (passengers identified as carrying illegal animal products and control). There were no significant effects for sex.

The highest frequencies of passengers bringing food of animal origin were in the months of June, July, August and September (Table 1), the holiday season in many countries, leading to a greater movement in the airports. Brazilian Central Bank data showed that, in 2011, there was a net increase in international travel expenses of US$ 14.7 billion, generating a 29.5% increase in expenses incurred by Brazilians abroad that effectively reached US $ 21.3 billion. Expenditures made by credit card by Brazilian tourists abroad that reached US $ 12.7 billion (Banco Central do Brasil 2011). This further increases the entry of international products into the Brazilian market.

**Table 1:** **Frequencies (F) and relative proportions (P) of passengers identified as carrying illegal animal products and control passengers per month at Guarulhos and Galeão international airports in Brazil**

	April	May	June	July	August	September	October	November	Total
PICRAP (F)	17	17	47	42	39	55	18	25	260
PICRAP (P)	0.07	0.07	0.18	0.16	0.15	0.21	0.07	0.10	
Control (F)	14	14	33	31	35	35	17	18	197
Control (P)	0.07	0.07	0.17	0.16	0.18	0.18	0.09	0.09	

PICRAP = Passengers identified as carrying illegal animal products.

One hundred forty-nine (149) passengers identified as carrying illegal animal products were found in Guarulhos and 111 in Galeão International Airport (p value of *χ*2 test was 0.0458). The frequency of passengers in this study was higher in Guarulhos, confirming Infraero statistics which recorded, from January to December 2010, a movement of 16,468,645 domestic and 10,380,540 international passengers in Guarulhos. In Galeão, in the same period, there were 9,210,885 domestic and 3,127,059 international passengers (Infraero Empresa Brasileira de Infraestrutura Aeroportuária 2012).

The age group most frequently bringing animal products (Table 2) was between 35 and 55 (p = 0.0207). The odds ratio comparing this group with all others was 1.64 (Confidence limits 1.12–2.42).

**Table 2:** **Frequencies (F) and proportions (P) of ages of passengers identified as carrying illegal animal products and control groups**

Age	PICRAP (F)	PICRAP (P)	Control (F)	Control (P)
**Group 1**: Less than 20 years	2	0.01	4	0.02
**Group 2**: Between 20 and 35 years	59	0.23	64	0.32
**Group 3**: Between 35 and 55 years	136	0.52	77	0.39
**Group 4**: Greater than 55 years	63	0.24	52	0.26
**Total**	260		197	

PICRAP = passengers identified as carrying illegal animal products.

This can be attributed in part to financial factors and this age group encompasses those economically active passengers, *i.e.* that part of the population financially independent and seeks to invest in travel to meet their personal needs. The economically active population comprises skilled manpower from the productive sector. Of a total of 101,110 people interviewed for the Yearbook of the Public System, Work and Income, 58,446 were between 30 to 59 years - the EAP (Economically Active Population) age group (Ministério do Trabalho e Emprego 2012).

Shih et al. (2005) studying the transport of products of animal origin evaluated the inspection of international air passengers arriving in Taiwan. Passengers that violated the regulations of the Bureau of Animal and Plant Health Inspection and Quarantine (BAPHIQ) were seen to be mainly of Chinese and Southeastern Asian origin and male passengers represented a higher risk than female passengers.

In the present study, there was a numerically higher frequency of male passengers in the group of passengers identified as carrying illegal animal products (141) *vs* 119 females (p-value of *χ*2 test was p = 0.44). The nationality of the passengers identified as carrying illegal animal products group included those from Brazil, Europe, America, Asia, South America and Africa (Table 3). Looking at individual countries, the highest frequencies of passengers were from Brazil, China, Portugal, Italy and Argentina. There were significant differences between nationalities (P < 0.0001). There were a large number of different nationalities found in the study, so the observations were divided into regions or continents to facilitate understanding. The area under the ROC curve was 0.6624 for nationality. It was observed that the greatest probability of entry with animal products were primarily from Eastern Europe (Romania and Turkey), followed by Asia and Latin Europe.

**Table 3:** **Point of origin, nationality and residency of passengers identified as carrying illegal animal products entering Brazil**

	Africa	Asia	Brazil	Eastern Europe	Latin Europe	Middle East	North America	Oceania	Russia	South Amercia	Western Europe	
	**Point of origin**											**Total**
**PICRAP**	16	35_a_	26	11_a_	78	8	15	0	0	55_a_	16_a_	260
**Control**	10	22_b_	27	1_b_	60	5	14	3	4	28_b_	23_b_	197
**Total**	26	57	53	12	138	13	29	3	4	83	39	457
	**Nationality of passenger**											**Total**
**PICRAP**	17	43_a_	91_a_	10_a_	50_a_	7	9		0	26_a_	7	260
**Control**	8	14_b_	114_b_	0_b_	20_b_	3	9		4	16_b_	9	197
**Total**	25	57	205	10	70	10	18		4	42	16	457
	**Domicile of passenger**											**Total**
**PICRAP**	10	14	144	10_a_	40_a_	2	11		0	24_a_	5_a_	260
**Control**	8	7	117	0_b_	21_b_	3	10		4	16_b_	11_b_	197
**Total**	18	21	261	10	61	5	21		4	40	16	457
	**Provenance of plane**											**Total**
**PICRAP**	19	31_a_		3	107_a_	9	17		0	56_a_	16	260
**Control**	14	17_b_		3	85_b_	11	17		1	36_b_	15	197
**Total**	33	48		6	192	20	34		1	92	31	457

Africa: South Africa, Angola, Egypt, Morocco; North and Central America: Canada, Costa Rica, Cuba, United States, Mexico, Panama, Puerto Rico; South America: Argentina, Bolivia, Chile, Colombia, Peru, Uruguay, Venezuela; Asia: China, Korea, India, Japan, Taiwan; Brazil: Brazil; Latin Europe: Spain, France, Italy, Portugal; Eastern Europe: Romania, Turkey; Oceania: Australia; Western Europe: Germany, Holland, Hungary, Norway, Poland, United Kingdom, Switzerland; Middle East: Qatar, United Arab Emirates, Israel, Iran, Iraq, Israel, Lebanon, Turkey; Russia: Russia and its borders Lithuania and Ukraine.

^a^ and ^b^ = Means in the same column followed by different letters are different (P < 0.05).

PICRAP = Passengers identified as carrying illegal animal products.

Brazilians were the largest group of passengers identified as carrying illegal animal products, followed by China, Canada, Italy and Argentina. This may reflect a lack of information about the entry ban into national territory of products from other countries of animal origin, as many people bring these products as a form of remembrance or as a present to loved ones. Probably, a lack of resources prohibits the interception all international flights and inspection all baggage by border staff.

The second most frequent country was the China. Portugal and Italy also showed a large movement of people. Many Brazilian descendants of Portuguese origin still travel to Portugal and return to Brazil with, for example, Portuguese Bacalhau (dried salted cod) without a valid animal health certificate. This food, even if certified abroad, cannot enter Brazilian territory without approval of the Brazilian Ministry of Agriculture, Livestock and Food Supply.

The point of departure (origin) of the passenger before arriving in Brazil was also important. The passengers identified as carrying illegal animal products group originated mainly in Latin Europe, followed by South America and Asia, (*χ*2 value 67.0484 and p = 0.0181). The area under the ROC curve was 0.6970, showing the importance of this factor. This large amount of passengers arriving from Latin Europe can be explained by the fact that countries making up this area (Spain, France, Italy, Portugal) have close historical links with Brazil and several countries in South America. This also includes passengers who begin a trip in Europe, but, after arriving in Brazil, they may travel on to other countries of South America, using Brazil as a stopover. There was a higher frequency of passengers identified as carrying illegal animal products from Latin Europe but analyzing countries individually, Argentina was the most frequent.

The fact that most passengers identified as carrying illegal animal products took the plane to Brazil from Portugal and Argentina is understandable if one analyses Brazil’s relationship with these countries. Portugal has a historical relationship with Brazil. Brazilians and Portuguese also have benefits and facilities in each other’s country (in terms of travel permits, residence etc.), reflected by investments in these countries, a common language favoring cultural exchange and privileged access to airlines, including direct flights.

Residency refers to the location where the passenger has his/her residence. The order of frequency in the passengers identified as carrying illegal animal products group were: Brazil, Latin Europe, South America, Asia and North America. When analyzed individually the main countries were Brazil, Italy, Portugal, China and the United States (*χ*2 p = 0.0049). The ROC curve area was 0.5858, therefore not as specific as for nationality or origin. Once again the greater likelihood of passengers entering with animal products in Brazil was from Eastern European countries. Disinformation for those who reside in Brazil is evident as many complained that they did not receive suitable information to avoid the entry of illegal products in the country.

Argentina, with 1.59 million visitors, had the highest number of South American landings in Brazil and Argentinians are the largest number of tourists to Brazil (Ministério do Turismo 2012). It must also be remembered that there are a large number of Argentines living in Brazil who visit their home country bringing typical products such as milk based sweets as souvenirs, gifts or to preserve their home culture.

Most passengers identified as carrying illegal animal products were travelling alone or with family (Table 4). The *a priori* reasons which lead to people traveling alone bringing more animal products than those who travel in groups is not known. Most people travel alone. According to the website of the magazine “National Geographic”, in 2009, 22.2 million of the 170 million Americans took a trip by themselves. Travel companies have been specializing in tours for those who want to travel unaccompanied (Loftus 2012). In the present study most passengers identified as carrying illegal animal products were traveling for tourism (95%) rather than work (5%).

**Table 4:** **Types of escort of the passengers identified as carrying illegal animal products**

	Friends	Couple	Excursion	Family	Alone	Total
**Frequency**	4	3	3	85	165	260
**Percentage (%)**	1.54	1.15	1.15	32.69	63.46	100.00

Another characteristic analyzed only in passengers identified as carrying illegal animal products group was the amount of baggage and the type of baggage. The results show that most people brought bags as baggage. The largest values for the quantity of baggage were respectively 3, 1, 2 and 4 volumes (Table 5). These were mainly suitcases and bags of some sort (Table 6).

**Table 5:** **Frequencies amounts of baggage brought by passengers identified as carrying illegal animal products**

Number of items of baggage											
	**1**	**2**	**3**	**4**	**5**	**6**	**7**	**8**	**9**	**12**	**Total**
**Freq.**	67	65	76	31	8	4	3	1	3	2	260
**%**	25.77	25	29.23	11.92	3.08	1.54	1.15	0.38	1.15	0.77	100

Freq.: Frequency.

**Table 6:** **Type and quantity of baggage carried by the passengers identified as carrying illegal animal products**

Type	Quantity
Bag	2
Box	2
Suitcase	157
Suitcase/Bag	30
Suitcase/Bag/Box	2
Suitcase/Bag/Box/Plastic bag	2
Suitcase/Bag/Box/Briefcase	1
Suitcase/Bag/Rucksack	6
Suitcase/Bag/Plastic bag	1
Suitcase/Bag/Briefcase	2
Suitcase/Box	6
Suitcase/Rucksack	35
Suitcase/Plastic bag	2
Suitcase/Thermal bag	1
Suitcase/Briefcase	5
Rucksack	2
Rucksack/Box/Briefcase	2
Rucksack/Plastic bag	1
Thermal bag	1

In a study developed in Australia about the pig producers’ perceptions of the Influenza Pandemic H1N1/09 outbreak and its effect on their biosecurity practices only 9.8% of those interviewed believed that that airport security, control of entry and quarantine measures applied were the main strength of the outbreak management (Hernández-Jover et al. 2012). This is similar to the situation found in Brazil, which shows that the population needs to be made aware of the dangers of introducing infectious agents by animal products in their baggage.

The risk of animal products being contaminated with transboundary infectious agents has been observed, showing the highest isolation risk of *Listeria monocytogenes* in marinated fish and lowest in cured- and dried-meat products exported and imported from Switzerland (Jemmi et al. 2002). Pig meat (Pharo and Cobb 2011), poultry (Cobb [Bibr CR11][Bibr CR12]) and the trade of small ruminants (Sherman 2011) and aquatic animals microbial (Rodgers et al. 2011) and their products have been shown to contain infectious agents and disseminate important diseases.

The rise in aircraft movement increases the chance of introduction of infectious agents by animal products or plants, as well as by insects present in baggage (Liebhold et al. 2006) and in aircraft. Since 1984, researchers have warned that the increase in air travel and the volume of air freight had considerably increased the risk of introduction of a foreign animal disease in most areas of the world (Sutmöller 1984). In Sydney International Airport, a research detected *Plum pox potyvirus* (PPV) in an illegal consignment of plum budwood and fruit intercepted by Australian Quarantine and Inspection Service (AQIS) inspectors (Davi et al. 2002). PPV was detected in leaf, bark and peduncle tissue. Recently, another study in Sydney International Airport, evaluated the possibility of introduction of a new West Nile Virus (WNV) strain in the country through mosquitos via international aircraft from United States, and concluded that the risk of introduction and spread of an exotic strain of WNV from the U.S via aircraft was low (Hernández-Jover et al. 2013).

Considering that one of the control measures to prevent introduction of exotic pathogens to free areas is to disinfect footwear of selected airplane passengers who have had contact with livestock or livestock premises while traveling abroad, researchers in the U.S. compared the effectiveness of the current USDA footwear disinfection protocol to a novel protocol and recommended implementation of the novel footwear disinfectant protocol (Amass et al. 2005).

A study in Japan also confirms the importance of surveillance in the borders (Taniguchi et al. 2008). Data from the National Epidemiological Surveillance for Infectious Diseases in humans were summarized from 1999 to 2008 on the situation of imported infectious diseases in Japan and observed that there is no border for infectious diseases. Diseases such as shigellosis, amebiasis, enterohemorrhagic *Escherichia coli* infection, acquired immunodeficiency syndrome, malaria, typhoid fever, dengue fever, giardiasis, cholera, hepatitis A and paratyphoid fever are important imported diseases and the introduction of new pathogens may result in their establishment in the country.

International transport can cause the dissemination of diseases such as Bovine spongiform encephalopathy (BSE), through live animal transport, waste products, when infected feed for pigs/poultry or pets is accidentally fed to cattle, or cross-contaminates as well as cattle feed handled or stored with meat-and-bone meal (Matthews and Adkin 2011).

Illegal importation of game from African countries seized at Paris Roissy-Charles de Gaulle airport was also seen to pose a serious risk. The illegal trade was estimated at five tonnes a week in this airport alone (Chaber et al. 2010). As well as the conservation question, wild animals and their products may disseminate diseases such as Ebola haemorrhagic fever virus, Severe Acute Respiratory Syndrome (SARS), monkeypox, Nipah and Hendra viruses, as well as West Nile virus. There has been a worldwide increase in the occurrence of these diseases recently, as well as tuberculosis, rabies and brucellosis (Travis et al. 2011).

Eggs from poultry for incubation may disseminate diseases such as Highly Pahonogenic Avian Influenza, Newcastle Disease and Aviarian Micoplasmosis (*Mycoplasma gallisepticum* or *M. synoviae*) (Cobb 2011a). Poultry meat may also contain up to 100 diseases such as Avian influenza, Infectious bursal disease, Newcastle disease, Turkey rhinotracheitis, Marek’s disease, Avian infectious bronchitis among others (Cobb 2011b).

Honey and its products may disseminate pathogens such as *Aethina tumida*, *Nosema ceranae*, *Varroa destructor* and *Paenibacillus larvae.* According to Mutinelli (2011), these can destroy bee colonies in countries why they have not previously existed. Pork and its byproducts may contain diseases such as foot and mouth disease, African swine fever, classical swine fever and swine vesicular disease (Pharo and Cobb 2011).

The transport of illegal small ruminants and their products may disseminate important diseases such as Foot and Mouth Disease, Rift Valley fever, Crimean Congo hemorrhagic fever, brucellosis and listeriosis (Sherman 2011). In aquatic animals and their products diseases include *Aerococcus viridans, Xenohaliotis californiensis, Yersina ruckeri*, Infectious haematopoietic necrosis virus, White spot syndrome virus etc., which may result in serious financial losses to shrimp, oyster and fish farms (Rodgers et al. 2011).

Liebhold et al. (2006) characterized species of insects transported by passengers in international airports in the United States. Between 1984 and 2000, 290,101 interceptions of foreign insects were made (15,000 per year). These may cause serious damage, for example to fruit or vegetable production, and the authors recommended a thorough and rigorous inspection to avoid the introduction of new diseases into the country.

This risk cannot be overlooked by the authorities who need to invest in research to enhance the chance of detection of animal products in baggage to minimize the possibility of transboundary infectious agents entering Brazil. Therefore, identification of the profile of passengers and amount and type of baggage they carry can be useful tools for identifying suspects of possessing illegal animal products in their baggage at international airports, as is already being used at airports worldwide for drug detection, in addition to other tools such as ultrasound equipment (Meijer and Bots 2003) and/or detection by sniffer dogs (Gazit and Terkel 2003).

## Conclusion

The group of passengers identified as carrying illegal animal products with highest risk are aged between 35 and 55 years old travelling alone. They are more likely to be from Eastern European countries, coming on flights from Portugal. Considering that even in the face of information about the ban, passengers insist on taking these products such as meat, cheeses, fish etc. into the country, these profile informations are important to efficiently identify possible passengers carrying illegal animal products.
